# The association of care transitions measure-15 score and outcomes after discharge from the NICU

**DOI:** 10.1186/s12887-020-02463-5

**Published:** 2021-01-04

**Authors:** Amy M. Yeh, Ashley Y. Song, Douglas L. Vanderbilt, Cynthia Gong, Philippe S. Friedlich, Roberta Williams, Ashwini Lakshmanan

**Affiliations:** 1 Division of Neonatology, LAC+USC Medical Center, Keck School of Medicine, University of Southern California, Los Angeles, CA USA; 2 Fetal and Neonatal Medicine Institute, Division of Neonatal Medicine, Children’s Hospital Los Angeles, 4650 Sunset Boulevard, MS #31, Los Angeles, CA 90027 USA; 3 Keck School of Medicine, University of Southern California, Los Angeles, CA USA; 4 Department of Preventive Medicine, Johns Hopkins University, Baltimore, MD USA; 5 Division of General Pediatrics, Children’s Hospital Los Angeles, Los Angeles, CA USA; 6 Leonard D. Schaeffer Center for Health Policy and Economics, University of Southern California, Los Angeles, CA USA; 7 Division of Cardiology, Children’s Hospital Los Angeles, Los Angeles, CA USA; 8 Department of Preventive Medicine, Keck School of Medicine, University of Southern California, Los Angeles, CA USA

**Keywords:** Neonate, Transition of care, Readmissions, Outcomes, Discharge, Quality, Social determinant of health, Early intervention, Quality of life, Limited English proficiency

## Abstract

**Background:**

Our objectives were (1) to describe Care Transitions Measure (CTM) scores among caregivers of preterm infants after discharge from the neonatal intensive care unit (NICU) and (2) to describe the association of CTM scores with readmissions, enrollment in public assistance programs, and caregiver quality of life scores.

**Methods:**

The study design was a cross-sectional study. We estimated adjusted associations between CTM scores (validated measure of transition) with outcomes using unconditional logistic and linear regression models and completed an E-value analysis on readmissions to quantify the minimum amount of unmeasured confounding.

**Results:**

One hundred sixty-nine parents answered the questionnaire (85% response rate). The majority of our sample was Hispanic (72.5%), non-English speaking (67.1%) and reported an annual income of <$20,000 (58%). Nearly 28% of the infants discharged from the NICU were readmitted within a year from discharge. After adjusting for confounders, we identified that a positive 10-point change of CTM score was associated with an odds ratio (95% CI) of 0.74 (0.58, 0.98) for readmission (*p* = 0.01), 1.02 (1, 1.05) for enrollment in early intervention, 1.03 (1, 1.05) for enrollment in food assistance programs, and a unit change (95% CI) 0.41 (0.27, 0.56) in the Multicultural Quality of Life Index score (*p* < 0.0001). The associated E-value for readmissions was 1.6 (CI 1.1) suggesting moderate confounding.

**Conclusion:**

The CTM may be a useful screening tool to predict certain outcomes for infants and their families after NICU discharge. However, further work must be done to identify unobserved confounding factors such as parenting confidence, problem-solving and patient activation.

## Background

Transition from the neonatal intensive care unit (NICU) to home can be a challenging time for families not only because of significant medical and developmental follow-up [1], but because of the high risk of readmission, particularly among preterm infants [2, 3]. Nationwide, readmission remains a substantial clinical and public health problem especially in the growing number of infants admitted and discharged from the NICU [4]. Harrison et al. described an overall increase in NICU admission rates from 2007 to 2012 from 64.0 to 77.9 per 1000 live births, almost 20%. This increase in admission rates spans all birth weight categories [5]. Similarly, Ray et al. have found an approximately 3-fold increase in risk of hospital readmission after discharge among premature infants compared to term infants. This increase in readmissions is inversely proportional to gestational age [2, 3, 6]. Moreover, Underwood et al. have found at least one readmission in 15% of preterm infants within the first year of life and infants < 25 weeks gestation had the highest rate of readmission, 31% [7]. Post-discharge, premature infants have higher health care costs and utilization including frequent pediatric outpatient visits and prescription medications [8]. It has been estimated the average cost per readmission is approximately $8500 and the average annual total cost in excess ranging approximately $41–93 million [2].

Preterm infants have extended lengths of stay in the NICU. Families build relationships with the physicians, nurses, and ancillary staff while in the NICU. The idea of discharge to home is at times only a hope but when it becomes a reality can be a vulnerable time for the families. Transition from the NICU to home may have tremendous impact on the infant, family, and society. Preparation for discharge from the NICU is critically important [9]. There are limited screening tools to evaluate this transition. In the pediatric population, Care Transitions Measure (CTM) [10] is a validated instrument most commonly used to assess the quality of care transition from outpatient pediatrics to outpatient adult medicine for adolescents and children with chronic medical problems. CTM has also been used to assess the quality of care transition after hospital discharge in the pediatric population [11, 12]. CTM has also been used to assess an intervention to improve care transition from NICU to ambulatory care [13]. While CTM has been used to investigate the association of quality of care transition in the outpatient setting, there are very limited studies using CTM to assess the quality of care transition after NICU discharge and examining the association between quality of care transition and readmission [14].

There is limited knowledge about the use of the CTM and health outcomes for infants discharged from the NICU and their families. Our objectives were (1) to describe the Care Transitions Measure scores among the caregivers in a population of preterm infants after discharge from the NICU and (2) to describe the association of the Care Transitions Measure scores with readmissions, enrollment in public assistance programs, and quality of life for the caregiver in a population of preterm infants after discharge from the NICU.

## Methods

### Study design and participants

The study design was a single-center, cross-sectional study. One caregiver of preterm (< 37 weeks’ gestation) infants up to 24 months corrected age with completed developmental assessments attending a high-risk infant follow-up clinic at a quaternary urban children’s hospital between 2013 and 2015 was enrolled. A 150-item questionnaire developed for this study was administered to participants about life after discharge from the NICU (Supplemental Material). Patient recruitment, survey administration and population characteristics are detailed in previous work [15, 16]. The Institutional Review Board at the center approved the study protocol. Written informed consent was obtained from all participants.

### Predictors: care transitions measure

We assessed quality of care transition using the Care Transitions Measure (CTM). CTM was first described by Coleman et al. CTM is a 15-item uni-dimensional measure of the quality of preparation for care transitions [10, 17–19]. CTM was found to have high internal consistency, reliability, and reflect 4 focus group-derived content domains [10]. CTM provides patient-centered insight into the quality of care transitions (questionnaire and coding is available in supplemental material). This score reflects the quality of the care transition with lower scores indicating a poorer quality transition and higher scores indicating a better transition. Responses are coded as, “Strongly Disagree,” “Disagree,” “Agree,” and “Strongly Agree.” Missing responses are accounted for and mean values are calculated for summated scores and subsequently a linear transformation is completed to create a CTM score of 0–100. There is an association between CTM scores and unwanted utilization outcomes (subsequent emergency department visit or re-hospitalization). CTM has been shown to discriminate between patients discharged from the hospital who did and did not have a subsequent emergency department visit or re-hospitalization for their index condition. This is useful information to clinicians, hospital administrators, quality improvement entities, and third party payers.

### Outcome: readmission

We assessed readmissions within the questionnaire administered to the parents. Parents were asked “Since the child has been home, have there been any other hospital stays?” The timeline was any all-cause hospitalization within 12 months of discharge. We also asked families about the number of hospitalizations and reasons for hospitalization including dehydration, feeding problems, infection, apnea, injury, poor weight, or other.

### Outcome: enrollment in public assistance programs

Information about the family’s use of community based developmental resources (e.g. early intervention programs), food assistance programs (Special Supplemental Nutrition Program for Women, Infants and Children (WIC) or Supplemental Nutrition Assistance Program (SNAP)), and Social Security Insurance (SSI) and Temporary Assistance to Needy Families (TANF) after NICU discharge was obtained via yes/no questions on the questionnaire.

### Outcome: parental quality of life

We assessed parental quality of life using the Multicultural Quality of Life Index (MCQLI). MCQLI [20] was developed by Mezzich et al.to address comprehensiveness, self-ratedness, cultural sensitivity, practicality, and psychometric soundness. MCQLI has 10 items that cover key aspects from physical well-being to spiritual fulfillment. MCQLI is brief, culturally informed, easy to complete, reliable, internally consistent, and valid.

### Statistical analysis

The characteristics of the study population were described using means and proportions. The frequency of covariates (race/ethnicity, income level, maternal education, language, infant birth weight, infant gestational age, neonatal co-morbidities, use of medical equipment and post discharge diagnoses) were compared across CTM scores. *P*-values were derived using t-tests for two group comparisons. Multivariable logistic regression estimated the adjusted odds of readmissions, enrollment in public assistance programs, and multivariable linear regression estimated adjusted parental quality of life scores with CTM scores. In previous research validating the strong association of the CTM with readmissions by Goldstein, et al., the CTM scores were transformed for every 10 point change and thus reflected in our regression models [21]. The models were adjusted for confounders such as race/ethnicity, maternal education, primary language, neonatal co-morbidities, post discharge diagnoses and use of medical equipment. Beta coefficients (linear regression results) and odds ratios (ORs) with 95% confidence intervals (CIs) and two-sided *P*-values for individual variable categories are reported.

We then conducted an E-value analysis, which is a type of sensitivity analysis to evaluate unmeasured confounding and whether unmeasured confounding contributed to the observed effects [22]. As detailed in previous work conducted by our group [23], the E-value analysis addresses how much unmeasured confounding would have to be to negate the observed results. A low E-value suggests that the results could easily be nullified by a confounder. Conversely, a very high E-value relative to the point estimate may imply that the observed effect is in fact plausible, because the strength and association of the unmeasured confounder with the exposure group and outcome must be very high to negate the observed effect.” [23]

### Power calculation

A sample size of at least 169 with unequal groups achieves 99% power to reject the null hypothesis of equal means when the population difference in CTM scores is 10 with a SD of 10 with a significance level (alpha) of 0.05 using a two sided two sample equal variance t-test (summary statement generated in PASS).

All statistical analyses were carried out using SAS, v. 9.4 (SAS Institute, Cary, NC, USA). E-values were then calculated using the R package “EValue” provided by the E-value creators [24].

## Results

**Fig. 1:**
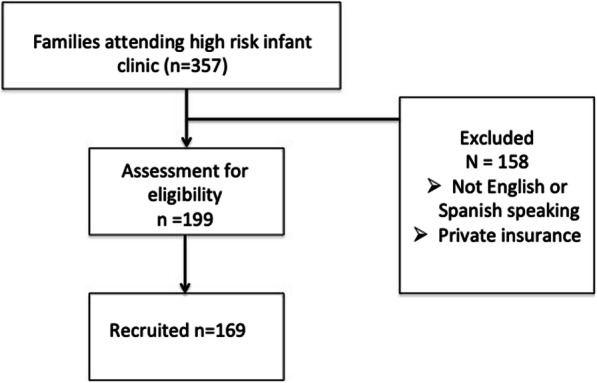
Flow chart of study population

There were 199 eligible participants. One hundred sixty-nine parents answered the questionnaire for an 85% response rate (Fig. 1). The majority of our sample was Hispanic (72.5%) and 58% of the sample had an annual income of <$20,000. About a third of our sample had an education level at high school or lower. 67.1% of the sample was non-English speaking. 80% of the infants had birth weight less than 1500 g. 61% of the infants had neonatal co-morbidities including at least one diagnosis of: fetal growth, surfactant deficiency, necrotizing enterocolitis, intraventricular hemorrhage grade 3 or 4, patent ductus arteriosus or retinopathy of prematurity. Nearly 29% of the infants used medical equipment post-discharge including oxygen, tracheostomy, wheelchair, adaptive stroller or feeding tube. 86.4% of the infants had post discharge diagnoses including at least one diagnosis of: autism, global developmental delay, cerebral palsy. All sample details are described in Table 1. The description of CTM score responses are available in Supplemental Table 1.

**Table 1:** Socio-demographics and infant characteristics with Care Transitions Measure score (*n* = 169)

		Care Transitions Measure Score	
	Total (N) (%)	Mean (SD)	*P* value
Socio-demographics			
Person completing survey			
Mother	156 (93.4)	79.3 (20.7)	0.09
Father	11 (6.6)	68.0 (29.6)	
Race/ethnicity			
White non-Hispanic	10 (6.5)	78.3 (20.4)	0.002
Hispanic	111 (72.5)	83.7 (17.5)	
Black non-Hispanic	16 (10.5)	66.0 (26.2)	
Other	16 (10.5)	72.9 (17.0)	
Income ($/Year)			
Less than $20,000	91 (58.0)	82.1 (19.2)	0.18
$20,001–$40,000	35 (22.3)	79.7 (23.2)	
$40,001–$60,000	14 (8.9)	70.0 (26.1)	
$60,001–$80,000	9 (5.7)	70.4 (23.5)	
More than $80,000	8 (5.1)	75.0 (9.8)	
Highest level of education (either parent)			
≤ High school	50 (36.2)	80.8 (19.1)	0.27
At least some college	88 (63.8)	76.7 (22.7)	
Language			
Non-English	104 (67.1)	80.8 (20.4)	0.39
English	51 (32.9)	77.7 (21.5)	
Infant characteristics			
Birthweight (grams)			
< 500 to < 1000	62 (53.0)	84.4 (18.3)	0.08
≥ 1000 to < 1500	32 (27.3)	84.4 (13.7)	
≥ 1500 to < 2500	16 (13.7)	94.5 (6.6)	
≥ 2500	7 (6.0)	79.3 (9.2)	
Gestational age (weeks)			
< 24 to < 28	56 (44.8)	83.3 (19.0)	0.08
≥ 28 to < 32	48 (38.4)	89.8 (10.8)	
≥ 32 to < 34	13 (10.4)	80.7 (14.1)	
≥ 34 to <37	8 (6.4)	80.3 (14.9)	
Neonatal co-morbidities^a^			
Yes	103 (61.0)	85.8 (16.4)	< 0.01
No	66 (39.0)	67.4 (23.5)	
Use of medical equipment^b^			
Yes	49 (29.0)	76.7 (22.7)	0.45
No	120 (71.0)	79.4 (20.9)	
≥ 2 clinic appointments/month			
Yes	124 (74.3)	77.2 (22.0)	0.05
No	43 (25.7)	84.3 (17.0)	
Post discharge diagnoses^c^			
Yes	108 (86.4)	84.6 (16.2)	0.18
No	17 (13.6)	90.1 (12.1)	
Enrolled in Early Intervention			
Yes	105 (62.9)	82.1 (18.9)	0.01
No	62 (37.1)	73.9 (23.4)	
Enrolled in food assistance programs^d^			
Yes	111 (70.3)	83.6 (17.0)	< 0.01
No	47 (29.7)	69.0 (25.5)	
Receiving Supplemental Security Income or Transitional Aid for Needy Families			
Yes	57 (33.7)	83.6 (18.7)	0.03
No	112 (66.3)	76.1 (22.3)	

Characteristics of neonates by survival are shown as mean (standard deviation). *P*-values derived using t-test (for 2 group comparison) and ANOVA test (for multi-group comparison)

^a^Neonatal co-morbidities include at least one diagnosis of: fetal growth restriction, surfactant deficiency, necrotizing enterocolitis, intraventricular hemorrhage grade 3 or 4, patent ductus arteriosus, retinopathy of prematurity

^b^Use of medical equipment includes: oxygen, tracheostomy, wheelchair, adaptive stroller, feeding tube

^c^Post discharge diagnoses include at least one diagnosis of: autism, global developmental delay, cerebral palsy

^d^Food Assistance Programs: Enrollment in the Special Supplemental Nutrition Program for Women, Infants and Children (WIC) or Supplemental Nutrition Assistance Program (SNAP)

Nearly 28% of the infants discharged from the NICU were readmitted. The number of re-hospitalizations and reasons were obtained (Supplement Table 2). The most common reasons for readmissions were breathing problems, dehydration or feeding problems. After adjusting for race/ethnicity, post discharge diagnoses, use of medical equipment, maternal education, primary language, and neonatal co-morbidities, we identified that a positive 10-point change of CTM score was associated with an odds ratio (95% CI) of 0.74 (0.58, 0.98) for readmission (*p* = 0.01) (Table 2). Interestingly, infants with a history of neonatal co-morbidities were less likely to be admitted, 0.34 (0.13, 0.88), *p* = 0.03. In addition, after adjustment, infants discharged home with medical equipment were 2.64 times more likely to be readmitted (95% CI 1.07, 6.5, *p* = 0.03). After adjustment, infants with post discharge diagnoses were 3.92 times more likely to be readmitted (95% CI 1.39, 11.06, *p* = 0.01).

**Table 2:** Association of change in the Care Transitions Measure score and at least one hospital readmission with E-value analysis (*n* = 169)

Effect	Estimate	95% Confidence Limits		*p*-Value	E-value	CI
Care Transitions Measure (for every10 point change in score)	0.74	0.58	0.98	0.01	1.6	1.1
Ethnicity						
Hispanic	0.61	0.21	1.81	0.37	1.89	1
Non-Hispanic	Reference					
Highest level of education (either parent)						
≤ High school	Reference					
At least some college	0.58	0.23	1.49	0.26	1.95	1
Language						
Non-English	1.91	0.68	5.39	0.22	2.11	1
English	Reference					
Neonatal co-morbidity^a^						
Yes	0.34	0.13	0.88	0.03	2.84	1.33
No	Reference					
Use of medical equipment^b^						
Yes	2.64	1.07	6.52	0.03	2.64	1.23
No	Reference					
Post-discharge diagnosis^c^						
Yes	3.92	1.39	11.06	0.01	3.37	1.64
No	Reference					

^a^Neonatal co-morbidities include at least one diagnosis of: fetal growth restriction, surfactant deficiency, necrotizing enterocolitis, intraventricular hemorrhage grade 3 or 4, patent ductus arteriosus, retinopathy of prematurity

^b^Use of medical equipment includes: oxygen, tracheostomy, wheelchair, adaptive stroller, feeding tube

^c^Post discharge diagnoses include at least one diagnosis of: autism, global developmental delay, cerebral palsy

The E-values were calculated using odds-ratios from our multivariable model and are also presented in Table 2. Specifically, we found a low E-value for the CTM score with an E-value of 1.60 (CI 1.1). This suggests that the change in CTM score may not explain lower odds of readmission despite an OR of 0.74 (*p* = 0.01). For other co-variates with a significant *p*-value such as neonatal co-morbidities, use of medical equipment and post-discharge diagnoses, we found moderate E-values of 2.84 (CI 1.33) for neonatal co-morbidities, 2.64 (CI 1.23) for use of medical equipment and 3.37 (CI 1.64) for post discharge diagnoses. These E-values suggest a moderate unmeasured confounder could explain differences in readmission for these examples.

**Fig. 2:**
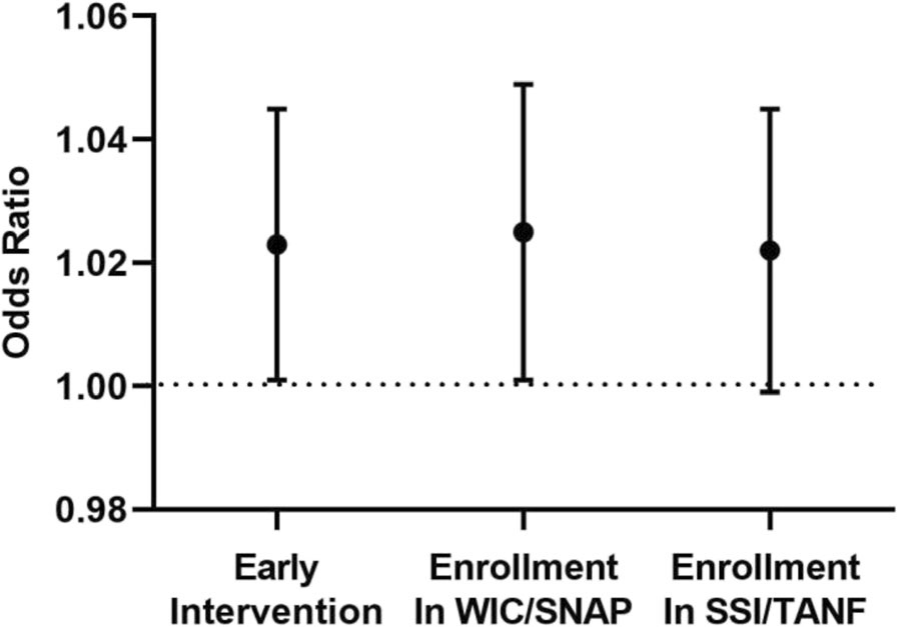
Adjusted association of change in Care Transitions Measure score (10 points) and enrollment in public assistance programs (early intervention, food assistance and supplemental security income/Transitional Aid to Needy Families) (*n* = 169). Legend: Models adjusted for race, English language proficiency, neonatal co-morbidities^a^, use of medical equipment^b^ and post-discharge diagnoses^c^. ^a^Neonatal co-morbidities include at least one diagnosis of: fetal growth restriction, surfactant deficiency, necrotizing enterocolitis, intraventricular hemorrhage grade 3 or 4, patent ductus arteriosus, retinopathy of prematurity. ^b^Use of medical equipment includes: oxygen, tracheostomy, wheelchair, adaptive stroller, feeding tube. ^c^Post discharge diagnoses include at least one diagnosis of: autism, global developmental delay, cerebral palsy

62.9% of families were enrolled in early intervention with a mean (SD) CTM score of 82.1 (18.9). 70.3% of families were enrolled in food assistance programs with a mean (SD) CTM score of 83.6 (17). 33.7% of the families were enrolled in SSI or TANF with a mean (SD) CTM score of 83.6 (18.7). We found an unadjusted association of higher CTM scores and enrollment in early intervention (*p* = 0.01), enrollment in WIC/SNAP (*p* < 0.001), and enrollment in SSI or TANF (*p* = 0.03). As depicted in Fig. 2, after adjusting for race/ethnicity, post discharge diagnoses, use of medical equipment, primary language, and neonatal co-morbidities, we identified that a positive 10-point change of CTM score was associated with an odds ratio (95% CI) an odds ratio (95% CI) of 1.02 (1, 1.05) for enrollment in early intervention and an odds ratio (95% CI) of 1.03 (1, 1.05) for enrollment in food assistance programs. This association between a positive 10-point change of CTM score and enrolment in SSI or TANF however after adjustment was not significant.

The mean Multicultural Quality of Life Index score for the sample was 80 and SD was 19.8. After adjusting for race/ethnicity, post discharge diagnoses, use of medical equipment, neonatal co-morbidities, and enrollment in early intervention, we identified that a positive 10-point change of CTM score was associated with an score change (95% CI) a unit change (95% CI) 0.41 (0.27, 0.56) in the Multicultural Quality of Life Index score (*p* < 0.0001) (Table 3).

**Table 3:** Adjusted association of change in Care Transitions Measure and Parental Quality of Life Scores^a^ (*n* = 169)

Parameter	Estimate	95% Confidence Limits		*p*-value
Care Transitions Measure (for every 10 point change in score)	0.41	0.27	0.56	< 0.0001
Race/Ethnicity				
White non-Hispanic	Reference			
Hispanic	4.47	−5.05	14	0.35
Black non-Hispanic	3.45	−8.29	15.2	0.56
Other	−7.24	−18.71	4.24	0.21
Neonatal co-morbidity^b^				
Yes	8.27	2.75	13.8	0.004
No	Reference			
Use of medical equipment^c^				
Yes	−4.97	−10.22	0.23	0.06
No	Reference			
Post-discharge diagnosis^d^				
Yes	1.91	−3.69	7.52	0.5
No	Reference			

^a^Parental quality of life scores represented by Multicultural Quality of Life scores

^b^Neonatal co-morbidities include at least one diagnosis of: fetal growth restriction, surfactant deficiency, necrotizing enterocolitis, intraventricular hemorrhage grade 3 or 4, patent ductus arteriosus, retinopathy of prematurity

^c^Use of medical equipment includes: oxygen, tracheostomy, wheelchair, adaptive stroller, feeding tube

^d^Post discharge diagnoses include at least one diagnosis of: autism, global developmental delay, cerebral palsy

## Discussion

We found a positive change in Care Transitions Measure (CTM) scores was associated with lower odds of readmission, higher odds of enrollment in public assistance programs and a positive unit change in parental quality of life scores for the caregiver in a population of preterm infants after discharge from the NICU.

Preterm infants are at 3–4 risk for readmissions compared to their term counterparts and hospitalizations incur up to $41 million annually in California [4]. In our study, 28% of infants discharged from the NICU were readmitted; this is consistent with previous literature by Underwood et al. [7] The CTM-15 has been used by the Hospital Consumer Assessment of Healthcare Providers and Systems patient satisfaction survey as a quality metric for transition of care. Previous studies have validated the strong association of the CTM with discharge success and readmissions in the adult population [17–19, 21]. There have been some concerns with the CTM-3, a 3 item version of the CTM-15 in predicting readmission because of its ceiling effect and generalizability to diverse populations [25]. In the pediatric population, CTM has most commonly been adapted to assess the quality of care transition from outpatient pediatrics to outpatient adult medicine for adolescents and children with chronic medical problems. It also has been used to assess the quality of care transition after hospital discharge in the pediatric population [11, 12]. Berry et al., described the association of parental perception of their child’s health at discharge and the risk of a subsequent unplanned readmission using CTM [26]. Previous studies highlight the importance of care transition for families of infants discharged from the NICU with complex medical needs [27, 28]. There seems to be a distinct discrepancy between parental views of care transition from health care professionals’ perceptions [29, 30]. There are very limited studies using CTM-15 to assess the quality of care transition after NICU discharge and examining the association between quality of care transition and readmission. The E-value analysis suggested that only a minimal unobserved confounder could contribute to the results. Possible unobservable constructs could include caregiver capacity such as problem solving, patient activation, health literacy or parenting confidence [31–34]. Moreover, medical complexity may also be another unobserved confounder such as infants with severe bronchopulmonary dysplasia or needing durable medical equipment [35]. Expectedly, infants with use of medical equipment and post-discharge diagnoses were associated with an increased odds of readmission [4, 36]. However, children with neonatal co-morbidities were *less* likely to be readmitted. The E-value analysis suggested that a moderate confounder could explain these results. For example, perhaps these families were exposed to better care-coordination tools and resources during their index hospitalization [37].

The American Academy of Pediatrics (AAP) recommends early intervention (EI) referrals for children who have or are at increased risk for developmental delays. Nationally in 2017, more than 370,000 infants and toddlers received EI services [38]. Despite a steady increase in the percentage of children obtaining services, many children who have or are at risk for developmental delays fail to receive EI services. Barfield et al. found that low-income and minority children may have more trouble accessing services [39]. Consistent with those findings, only 62.9% of eligible children were enrolled in EI in our sample. This discrepancy in enrollment is a disadvantage and is seen greater in the low-income and minority children [40]. There is an increasing need for better connection with community based programs. Also, an improvement in the Care Transitions Measure score was only marginally associated with enrollment in early intervention. Perhaps, we need better assessment tools at discharge for predicting successful enrollment in EI than just the CTM-15.

We showed a positive change in CTM scores was associated with lower risk of a poor parental quality of life for the caregiver. There are few studies assessing parental quality of life after their infants are discharged from the NICU. Most of the studies assess health-related quality of life (HRQOL) in the adult population. A recent study by our group described the impact of preterm birth on parents and families and identified associated modifiable factors [41]. We also identified that access to email, text messaging, and smartphones was associated with higher parental quality of life [15]. In addition McAndrews et al. also described an association between extremely preterm infants and worse HRQOL during the NICU hospitalized but with more improvement after discharge than others hospitalized in the NICU. The study further elaborates that a complex home care after discharge was associated with lower parent HRQOL [42]. The CTM may provide a screening measure for further exploration of family quality of life around discharge.

Our study is novel in exploring the Care Transitions Measure as a screening tool for infant/family outcomes in a population of preterm infants after discharge from the NICU. However, it is prone to measurement bias and selection bias. The parents in our study may have had recall bias when answering the questionnaire after discharge from the NICU. Response bias is also an important limitation of patient satisfaction surveys. Mazor et al. described patients who were more satisfied with their care were more likely to respond to patient satisfaction surveys leading to inflated scores [43]. This could potentially lead to an underestimate of the association between low Care Transitions Measure scores with increased risk of readmission. Selection bias is possible when enrolling patients from a high risk infant follow up clinic at a quaternary urban children’s hospital which does not encompass all patients discharged from the NICU. The infants who qualify for the high risk infant follow up clinic but either did not follow up or had follow up in another clinic were not enrolled.

## Conclusion

The CTM-15 may be a useful screen in a population of preterm infants and their families to predict certain health outcomes. However, our sensitivity E-value analysis suggested that minimal-moderate confounding may affect our results. Future work should assess constructs such as caregiver self-efficacy, problem solving, patient activation and paucity of resources. By better understanding these kinds of risk factors, we might be able to assemble a more comprehensive risk stratification system for successful transitions after NICU discharge.

## Supplementary Information

**Additional file 1: Supplemental Table 1.** Distribution of Care Transitions Measure Score Items. **Supplemental Table 2.** Readmissions and Causes.

**Additional file 2.** Questionnaire and Scoring.

## Data Availability

The datasets used and/or analyzed during the current study available from the corresponding author on reasonable request.

## Abbreviations

CTM-15: Care Transitions Measure 15
NICU: Neonatal Intensive Care Unit
MCQLI: Multicultural Quality of Life Index
