# Supplementary material for: The association of care transitions measure-15 score and outcomes after discharge from the NICU
Source: BMC Pediatr. 2021 Jan 4;21:7. doi: 10.1186/s12887-020-02463-5 (PMC7780380; doi:10.1186/s12887-020-02463-5)
Supplement: Supplementary file 1 — Additional file 1: Supplemental Table 1. Distribution of Care Transitions Measure Score Items. Supplemental Table 2. Readmissions and Causes. [file 12887_2020_2463_MOESM1_ESM.docx]

**Supplemental Table 1. Distribution of Care Transitions Measure Score Items**

| Items | Question | Mean | SD | Minimum | Maximum |
| --- | --- | --- | --- | --- | --- |
| CTM-1 | Before I left the hospital, the staff and I agreed about clear health goals for me and how these would be reached | 3.29 | 0.80 | 1.00 | 4.00 |
| CTM-2 | The hospital staff took my preferences and those of my family or caregiver into account in deciding what my health care needs would be when I left the hospital | 3.28 | 0.80 | 1.00 | 4.00 |
| CTM-3 | The hospital staff took my preferences and those of my family or caregiver into account in deciding where my health care needs would be met when I left the hospital | 3.28 | 0.85 | 1.00 | 4.00 |
| CTM-4 | When I left the hospital, I had all the information I needed to be able to take care of myself | 3.37 | 0.77 | 1.00 | 4.00 |
| CTM-5 | When I left the hospital, I clearly understood how to manage my health | 3.40 | 0.79 | 1.00 | 4.00 |
| CTM-6 | When I left the hospital, I clearly understood the warning signs and symptoms I should watch for to monitor my health condition | 3.33 | 0.80 | 1.00 | 4.00 |
| CTM-7 | When I left the hospital, I had a readable and easily understood written plan that described how all of my health care needs were going to be met | 3.36 | 0.80 | 1.00 | 4.00 |
| CTM-8 | When I left the hospital, I had a good understanding of my health condition and what makes it better or worse | 3.43 | 0.71 | 1.00 | 4.00 |
| CTM-9 | When I left the hospital, I had a good understanding of the things I was responsible for in managing my health | 3.33 | 0.80 | 1.00 | 4.00 |
| CTM-10 | When I left the hospital, I was confident that I knew what to do to manage my health | 3.36 | 0.73 | 1.00 | 4.00 |
| CTM-11 | When I left the hospital, I was confident I could actually do the things I needed to do to take care of my health | 3.39 | 0.76 | 1.00 | 4.00 |
| CTM-12 | When I left the hospital, I had a readable and easily understood written list of the appointments or tests I needed to complete within the next several weeks | 3.38 | 0.77 | 1.00 | 4.00 |
| CTM-13 | When I left the hospital, I clearly understood the purpose for taking each of my medications | 3.46 | 0.73 | 1.00 | 4.00 |
| CTM-14 | When I left the hospital, I clearly understood how to take each of my medications, including how much I should take and when | 3.46 | 0.75 | 1.00 | 4.00 |
| CTM-15 | When I left the hospital, I clearly understood the possible side effects of each of my medications | 3.21 | 0.81 | 1.00 | 4.00 |

CTM- care transitions measure

**Supplemental Table 2. Readmissions and Causes**

| Post-discharge |  | Care Transitions Measure Score |  |
| --- | --- | --- | --- |
|  | Total | Mean (SD) | P value |
| Any other hospital stay |  |  |  |
| Yes | 48 | 72.8 (22.7) | 0.02 |
| No | 121 | 81.1 (20.5) |  |
| Child stayed in hospital-how many times |  |  |  |
| 1 | 25 | 76.1 (23.0) | 0.62 |
| 2 | 15 | 64.3 (29.2) |  |
| 3 | 6 | 68.2 (14.9) |  |
| 4 | 1 | 86.7 (0) |  |
| 5 | 1 | 68.9 (0) |  |
| Hospital stay reasons (all apply)* |  |  |  |
| Breathing problems | 20 | 73.0 (23.2) | NA |
| Dehydration | 3 | 50.4 (37.9) | NA |
| Feeding problems | 9 | 66.2 (21.1) | NA |
| Infection | 3 | 53.3 (13.3) | NA |
| Apnea | 4 | 67.2 (17.2) | NA |
| Injury | 4 | 36.1 (19.2) | NA |
| Poor weight gain | 1 | 66.7 (0) | NA |
| Other | 15 | 82.6 (19.8) | NA |
