# Supplementary material for: The association of care transitions measure-15 score and outcomes after discharge from the NICU
Source: BMC Pediatr. 2021 Jan 4;21:7. doi: 10.1186/s12887-020-02463-5 (PMC7780380; doi:10.1186/s12887-020-02463-5)
Supplement: Supplementary file 2 — Additional file 2. Questionnaire and Scoring. [file 12887_2020_2463_MOESM2_ESM.pdf]

CARE TRANSITIONS MEASURE (CTM-15)

Patient Name: ____ Date: _______________

Who completed interview? θ Patient θ Caregiver

The first few statements are about the time you were in the hospital . . .

1. Before I left the hospital, the staff and I agreed about clear health goals for me and how these would be reached.

Strongly Disagree	Disagree	Agree	Strongly Agree	Don't Know/ Don't Remember/ Not Applicable

2. The hospital staff took my preferences and those of my family or caregiver into account in deciding ***what*** my health care needs would be when I left the hospital.

Strongly Disagree	Disagree	Agree	Strongly Agree	Don't Know/ Don't Remember/ Not Applicable

3. The hospital staff took my preferences and those of my family or caregiver into account in deciding ***where*** my health care needs would be met when I left the hospital.

Strongly Disagree	Disagree	Agree	Strongly Agree	Don't Know/ Don't Remember/ Not Applicable

The next set of statements is about when you were preparing to leave the hospital . . .

4. When I left the hospital, I had all the information I needed to be able to take care of myself.

Strongly Disagree	Disagree	Agree	Strongly Agree	Don't Know/ Don't Remember/ Not Applicable

5. When I left the hospital, I clearly understood how to manage my health.

Strongly Disagree	Disagree	Agree	Strongly Agree	Don't Know/ Don't Remember/ Not Applicable

© Eric A. Coleman, MD, MPH, all rights reserved

6. When I left the hospital, I clearly understood the warning signs and symptoms I should watch for to monitor my health condition.

Strongly Disagree	Disagree	Agree	Strongly Agree	Don't Know/ Don't Remember/ Not Applicable

7. When I left the hospital, I had a readable and easily understood written plan that described how all of my health care needs were going to be met.

Strongly Disagree	Disagree	Agree	Strongly Agree	Don't Know/ Don't Remember/ Not Applicable

8. When I left the hospital, I had a good understanding of my health condition and what makes it better or worse.

Strongly Disagree	Disagree	Agree	Strongly Agree	Don't Know/ Don't Remember/ Not Applicable

9. When I left the hospital, I had a good understanding of the things I was responsible for in managing my health.

Strongly Disagree	Disagree	Agree	Strongly Agree	Don't Know/ Don't Remember/ Not Applicable

10. When I left the hospital, I was confident that I knew what to do to manage my health.

Strongly Disagree	Disagree	Agree	Strongly Agree	Don't Know/ Don't Remember/ Not Applicable

11. When I left the hospital, I was confident I could actually do the things I needed to do to take care of my health.

Strongly Disagree	Disagree	Agree	Strongly Agree	Don't Know/ Don't Remember/ Not Applicable

© Eric A. Coleman, MD, MPH, all rights reserved

The next statement is about your follow-up doctors' appointments . . .

12. When I left the hospital, I had a readable and easily understood written list of the appointments or tests I needed to complete within the next several weeks.

Strongly Disagree	Disagree	Agree	Strongly Agree	Don't Know/ Don't Remember/ Not Applicable

The next set of statements is about your medications…

13. When I left the hospital, I clearly understood the ***purpose*** for taking each of my medications.

Strongly Disagree	Disagree	Agree	Strongly Agree	Don't Know/ Don't Remember/ Not Applicable

14. When I left the hospital, I clearly understood ***how*** to take each of my medications, including how much I should take and when.

Strongly Disagree	Disagree	Agree	Strongly Agree	Don't Know/ Don't Remember/ Not Applicable

15. When I left the hospital, I clearly understood the possible ***side effects*** of each of my medications.

Strongly Disagree	Disagree	Agree	Strongly Agree	Don't Know/ Don't Remember/ Not Applicable

© Eric A. Coleman, MD, MPH, all rights reserved

Scoring the CTM®-15

Overall Quality of Care Transition Score: This score reflects the overall quality of the care transition, with lower scores indicating a poorer quality transition, and higher scores indicating a better transition.

Scoring Protocol

Step 1: Code responses as Strongly Disagree =1; Disagree =2; Agree =3; Strongly Agree =4.

Step 2: Assign code (e.g., 9) to missing responses, and a different code (e.g., 99) to Don't Know/Don't Remember/Not Applicable. These will not be counted as answered questions for Step 3a, as the 9 and 99 codes are not included in the 4 point Likert scale and therefore will not contribute to the CTM® score. You can, however, get a count of 99's in order to calculate a percentage of these responses relative to questions answered (step 3a.)

Step 3: Compute a mean score for each respondent based only on the questions answered. To do this:

- Step 3a: For each respondent count the number of questions answered. (In SPSS, Step 3a is accomplished with the Count command in the Transform menu and Step 3b by a Compute command).
- Step 3b: For each respondent obtain a summated score by adding Step 1 values across answered questions.
- Step 3c: Obtain **mean** for each respondent by dividing Step 3b result by Step 3a result. The name of this value is **mean**.

Step 4: Perform a linear transformation of the result of Step 3c to obtain a user-friendly 0-100 score. Use the following formula:

- 0-100 CTM® Score for each respondent = [(Step 3c result-1)/3]*100 .
- In SPSS Syntax this computation is:

```
COMPUTE CTM15_0_100 = (((ctm15)-(1))/(3))*100 .
EXECUTE .
```

© Eric A. Coleman, MD, MPH, all rights reserved
